# Occurrence of Dysphagia After Correction Surgery in the Cervical Spine for Dropped Head Syndrome

**DOI:** 10.7759/cureus.55067

**Published:** 2024-02-27

**Authors:** Hiroshi Miyamoto

**Affiliations:** 1 Orthopedic Surgery, Kobe Rosai Hospital, Kobe, JPN

**Keywords:** spine, dysphagia, complication, correction surgery, dropped head syndrome, kyphosis, cervical

## Abstract

Correction surgery for dropped head syndrome (DHS) is a challenging procedure that requires extensive realignment of the cervical spine and is associated with a high rate of complications. Postoperative occurrence of dysphagia related to the change of the cervical alignment is well known as a complication of occipito-cervical fusion, and it is thought to be caused by narrowing of the pharyngeal airway space (PAS) due to the change of the alignment. We experienced a case of severe dysphagia requiring tracheotomy and gastrostomy after correction surgery for DHS. Revision surgery which downgraded the cervical lordosis immediately solved this problem. We report this case and discuss the possible risk factors causing this complication.

## Introduction

Correction surgery for dropped head syndrome (DHS) is a challenging procedure that requires wide-ranged realignment of the cervical spine and is associated with a high rate of complications [1]. Postoperative difficulty in swallowing is a devastating complication that threatens life-support, similar to bladder and bowel disturbance. Generally, the occurrence of postoperative dysphagia is a well-known complication after anterior procedures of the cervical spine [2-5]. The possible risk factors for postoperative dysphagia after anterior procedures include intraoperative esophageal traction [2], long operative time [3], multilevel surgery [2], soft tissue swelling [4], cervical plate use [3], and recurrent laryngeal nerve damage [5]. Most cases of postoperative dysphagia or airway-related complications after surgery for DHS were observed in patients treated with combined (anterior and posterior) approaches [1].

It has been reported that postoperative dysphagia may also occur in patients who have undergone posterior realignment after occipito-cervical fusion (OCF) [6,7]. Reduction in the postoperative OC-2 angle compared to that of pre-operation is a risk factor for postoperative dysphagia caused by narrowing of the pharyngeal airway space (PAS) between the tongue root and the posterior pharyngeal wall [7]. Protrusion of the apex of cervical lordosis anterior to the S-line, a line passing through the center of the C1 anterior arch and perpendicular to McGregor’s line, is another risk factor for postoperative dysphagia caused by narrowing of the PAS [6].

We experienced a case of severe dysphagia after correction surgery for DHS. Revision surgery which downgraded the cervical lordosis immediately solved this problem. We report this case and discuss the possible risk factors causing this complication.

## Case presentation

A 68-year-old female had suffered chin-on-chest deformity for three years. No neurological deficit was found. She did not complain of any difficulty in swallowing preoperatively, and the preoperative videoendoscopic evaluation of swallowing (VE) score [8,9] was 1 (0-1-0-0).

**Figure 1:**
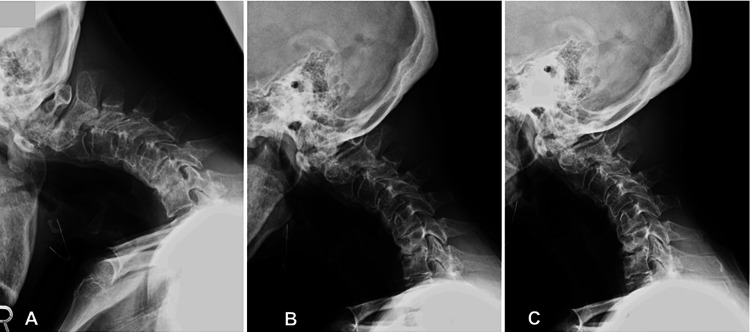
Preoperative flexion-extension lateral radiographs of a 68-year-old female patient with dropped head syndrome (A) Flexion, (B) neutral, and (C) extension

**Figure 2:**
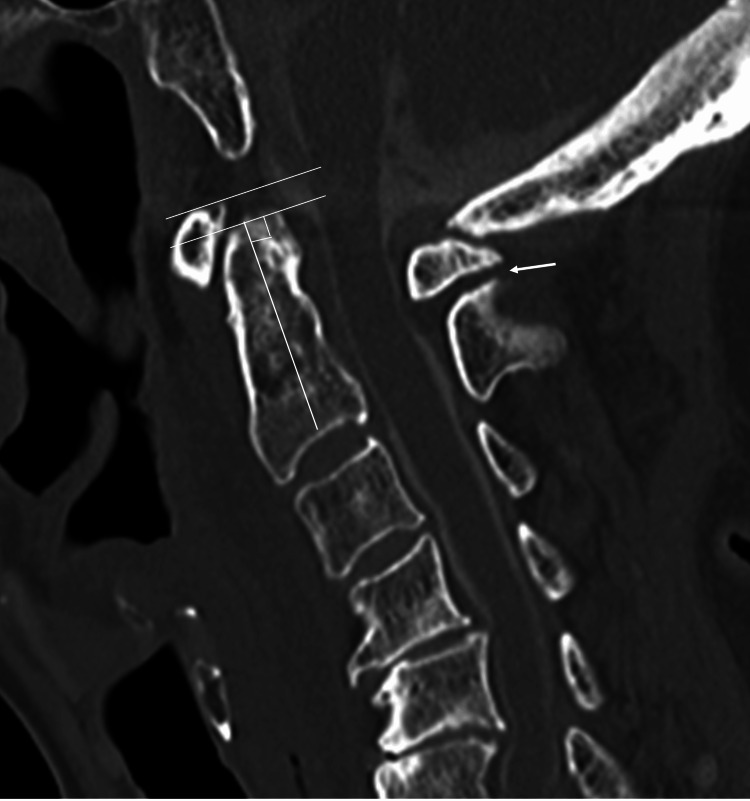
CT image showing the specific radiological findings between C1 and C2 C1 anterior arch exceeding the tip of the dens (white line) Pseudoarthrosis formation between the C1 posterior arch and C2 lamina (white arrow)

Flexion-extension radiography showed rigid cervical kyphosis with a C2-7 angle of -50 degrees (Figures 1A-1C). The uncovertebral joints and the facet joints between C2/3 and C5/6 were fused. Radiography failed to detect the hard palate because of the severe kyphotic deformity. The C1-2 angle was 50 degrees in the neutral position and little change in flexion and extension (49 and 53 degrees, respectively). Notably, the cephalad tip of the anterior arch of the axis exceeded the tip of the dens, and the caudal margin of the posterior arch of the axis came close to the cephalad margin of the C2 lamina, which appeared to be forming pseudoarthrosis (Figures 1A-1C, 2).

Initial treatment plan

**Figure 3:**
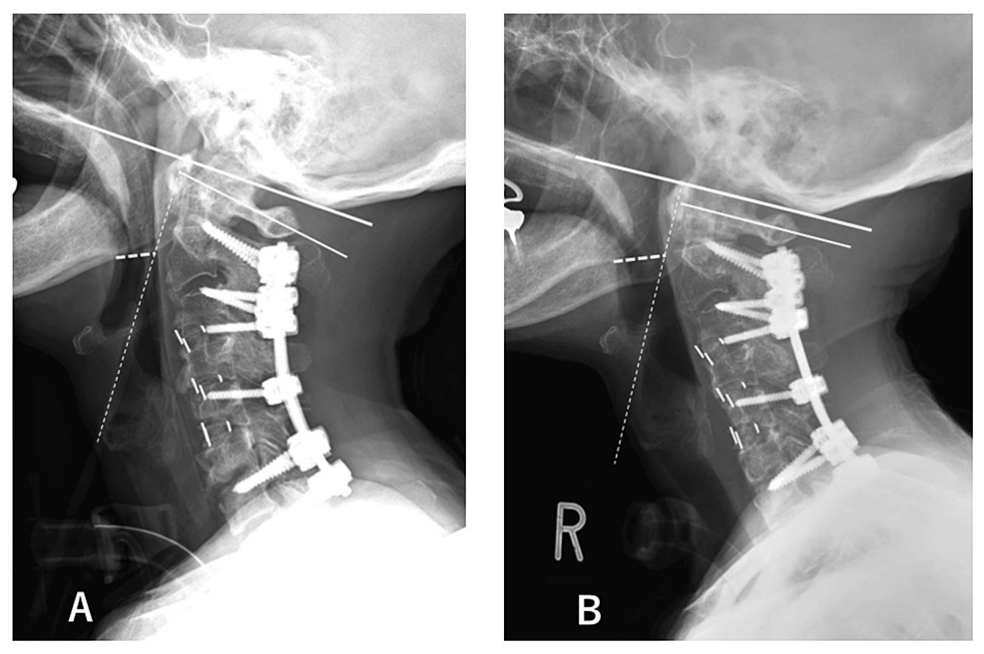
Lateral radiograph of the cervical spine after initial correction surgery (A) and after downgrading surgery (B) (A) Lateral radiograph after initial correction surgery: C2-7 angle was 20 degrees, C1-2 angle appeared hyperlordotic (45 degrees), and specific abnormal radiological findings included the anterior arch of the axis extending over the dens, osteophyte formation around the anterior arch of the axis and the dens, and pseudoarthrosis formation between the posterior arch of the axis and the lamina of C2). OC-1 angle was -12 degrees (white line). S-line was minus (small dotted white line). (B) Lateral radiograph after downgrading surgery. C2-7 angle changed to 10 degrees. C1-2 angle was 40 degrees. OC-1 angle increased to 2 degrees (white line). S-line was minus (small dotted white line). The narrowest PAS was increased between pre- and post-downgrading surgery (dotted white line). PAS: pharyngeal airway space

Conservative treatment, consisting of the application of a soft collar, active range of motion exercises, and administration of analgesics for two months [10], failed, and the patient agreed to surgical intervention. Three-stage surgery was performed as follows. First, a posterior procedure was performed including cervical pedicle screw insertion into the bilateral C2, C7, and T1, right-side C3, and left-side C4, and facetectomy at the bilateral C2/3/4/5/6. Second, anterior release and cage insertion at C3/4/5/6 were performed. Third, posterior correction using rod connection onto the pedicle screws and bone grafting were performed [11]. Operation time was 540 minutes, and the total amount of bleeding was 462 ml. Postoperative radiography showed that the C2-7 angle had increased to 20 degrees (Figure 3A). The S-line was minus [6].

Results

She remained in the intensive care unit without extubation on the day of surgery (day 0). The intubation tube was removed on day 1, but she could not swallow water. The postoperative VE score was 11 (3-2-3-3). She suffered aspiration pneumonia, so tracheotomy and gastrostomy were performed on day 21. However, her loss of swallowing function did not improve despite rehabilitation.

Second treatment plan

**Figure 4:**
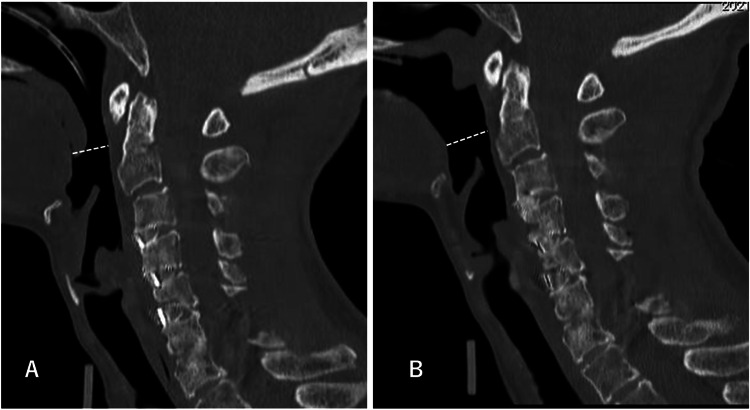
Mid-sagittal CT image after initial surgery (A) and after downgrading surgery (B) The narrowest PAS was increased from 10 mm (A, dotted white line) to 15 mm (B, dotted white line). PAS: pharyngeal airway space

After obtaining informed consent, downgrading surgery was performed consisting of rod removal, modification of the alignment, and new rod connection with moderate contour two months after the initial surgery. C2-7 angle was downgraded to 10 degrees (Figure 3B). To identify the cause of this devastating complication, the images taken pre- and post-downgrading surgeries were thoroughly compared (Figures 3A, 3B, 4A, 4B).

We found several differences as follows: (1) the C2-7 angle was 20/10 degrees, (2) the narrowest PAS (nPAS) was 10/15 mm (Figures 4A, 4B), and (3) the OC-1 angle between McGregor’s line and C1 was -12/2 degrees (Figures 3A, 3B) at pre-/post-downgrade, respectively. These measurements were obtained from the plain lateral radiographs except (2) which was measured on the mid-sagittal plane of the CT images.

Outcome

She could drink water and eat on the day after revision surgery. The tracheotomy and gastrostomy were closed three months after the revision.

## Discussion

DHS is a rare clinical entity defined as a chin-on-chest deformity in standing or sitting positions. Patients with DHS exhibit difficulty in holding their head and neck in neutral and extension positions. Consequently, the quality of life of patients with DHS is severely impacted due to difficulty in horizontal gaze, neck pain, cervical myelopathy, and cosmetic problems [12]. Therefore, surgical intervention can be applied when conservative treatment fails [10]. However, the requirement for extensive realignment of the cervical spine is associated with a high rate of complications [1]. Kurakawa et al. reported that correction and posterior fixation of cervical kyphosis may cause a high percentage having postoperative complications such as C5 nerve palsy [13].

Miyamoto et al. reported that the application of a formula such as T1 slope minus 20 degrees equivalent to postoperative C2-7 angle can provide acceptable clinical outcomes without any further additional surgeries for DHS [14]. In the present case, the postoperative C2-7 angle would be 7 degrees because the preoperative T1 slope was 27 degrees. However, this rigid cervical kyphosis due to the uncovertebral and facet joint fusions required three-level anterior/posterior release and cage insertion resulting in having unexpected “good” cervical lordosis although reacquisition of horizontal gaze was fully achieved.

Most cases of postoperative dysphagia or airway-related complications after surgery for DHS were reported to be related to anterior procedure, not the alignment change [1]. However, realignment was certainly related to the dysphagia in our patient because immediate relief of the symptoms occurred after downgrading surgery which decreased the cervical lordosis. We suggest that reduced nPAS can cause postoperative dysphagia after correction surgery for DHS of the middle/lower cervical spine.

The cranio-vertebral junction may provide a compensation mechanism when trying to obtain a horizontal gaze by allowing the upper cervical spine to become extremely hyperlordotic if the patient cannot gaze horizontally [15]. However, whether the OC-1 or C1-2 angle is more important in this compensatory mechanism remains unclear [7]. The preoperative C1-2 angle was large (50 degrees) in our present case, and the range further front and back was only 1 and 2 degrees, respectively. Moreover, specific radiological findings were observed to indicate contracture between C1 and C2, such as osteophyte formation between the C1 anterior arch and dens, C1 anterior arch exceeding the tip of the dens, and pseudoarthrosis formation between the C1 posterior arch and C2 lamina. In this case, the contracture at C1-2 might have resolved during the follow-up period. However, the severe dysphagia which required tracheotomy and gastrostomy and persisted for two months could not remain untreated. As a result, revision surgery was conducted before solid bony union occurred.

## Conclusions

In conclusion, we present a case of a 68-year-old female patient who suffered from postoperative severe dysphagia which was resolved by downgrading surgery. This is the first report of postoperative dysphagia due to the realignment of cervical kyphosis for DHS. Reciprocal change likely occurs at OC-1 rather than C1-2 in patients with DHS who underwent correction of kyphosis in the middle/lower cervical spine. That is, a reduction in the OC-1 angle can cause a decrease in nPAS resulting in postoperative dysphagia. Therefore, through this report, we aim to highlight that overcorrection of the cervical alignment should be avoided, especially in patients with severe degeneration and rigid kyphosis, possibly with contracture at C1/2 in the hyperlordotic alignment.
